# What do the narratives tell us? Exploring the implementation of the Athena SWAN Ireland Charter

**DOI:** 10.3389/fsoc.2022.1058397

**Published:** 2023-01-04

**Authors:** Monica O'Mullane

**Affiliations:** Marie Skłodowska-Curie Action Fellow, Institute for Social Science in the 21st Century (ISS21), University College Cork, Cork, Ireland

**Keywords:** gender equality, higher education, policy implementation, Ireland, Athena SWAN

## Abstract

Due to the systemic inequalities enduring in career progression pathways in the Irish higher education sector, the Athena SWAN Ireland Charter (ASIC), a gender equality accreditation program, is being implemented. Using a theoretical approach, blending insights from feminist institutionalism with literature on the role of narratives in policy implementation, this article reveals the complex nature of subjective engagement with policy implementation processes. This article discusses an empirical study of Athena SWAN Ireland Charter implementation across three purposively chosen Irish universities, interviewing 26 key institutional actors tasked with implementing the ASIC locally. Narrative themes emerging as dominant from the data include a lack of operational knowledge, desire for a nationally contextualized program, ambiguity, championing, “happy talk,” and identifying points of resistance. Literature on the role of narrative accounts highlighting a diversity of perceptions in policy and program implementation is strengthened by this study's findings. A feminist institutionalist lens highlight the gendered nature of the operationalization of the Charter work and the vague and detached “happy talk” engaged predominantly by senior men leaders. Findings from this empirical study highlight the importance of exploring the narrative accounts of key actors in order to gain a holistic understanding of the nuanced implementation process, beyond the normative assumptions inherent in the Charter implementation.

## Introduction

Irish higher education policy has endorsed the implementation of a structural change program—the Athena SWAN Ireland Charter (ASIC)—to improve gender equity within higher education institutions (HEIs). This move is a response to persistent inequalities in career progression trajectories in Irish HEIs, whereby just 25% of professors are women (HEA, 2020). This figure resonates with the experience across the European Union (EU), with the latest figures highlighting an average of 26% of women professors in a comparable time period (EC, 2021). Consensus in the literature affirms that academia is “stubbornly gendered as masculine” (Mackay, 2020; p. 77). In Irish HEIs, O'Connor (2020) concurs that the legacy of masculinist dominance plays a major role in inhibiting structural and cultural organizational changes. Unsurprisingly, research has shown that structural change programs in academic contexts often fail in challenging or transforming institutional processes and gendered norms (Van Den Brink and Benschop, 2012; Powell et al., 2018). Related to the Athena SWAN Charter as a structural change program, research shows that there is limited impact on addressing gender inequality through the Charter in the UK except in the minority Gold Award institutions (Graves et al., 2019).

Inspired by Henderson and Bhopal (2021) study of the narratives of academic staff involved in the Athena SWAN and Race Equality Charters in the UK, and in response to limited Irish data on how staff in HEIs perceive gender equality programs (Hodgins et al., 2022), this article is premised on one key question: could our understanding of the narratives of key institutional actors play a role in enhancing ASIC implementation? Key institutional actors are HEI staff who are members of their institutional Athena SWAN Ireland Charter Self-Assessment Teams (SATs). This questioning is grounded in the reality that such program implementation does not happen normatively in a rational, linear manner, meaning that “the actions and commitments (of ASIC) are not simply installed into the technical rules and procedures of HEIs, resulting in the organizational and cultural change it seeks” (O'Mullane, 2021, p. 1). ASIC implementation is facilitated by key institutional actors—HEI staff—who possess their subjective interpretations, understandings, and ideas, contributing to a complex and non-linear process of implementation (Ball, 2013). Therefore, the purpose of this inquiry is also to gain a deeper understanding of the reality of the policy implementation process beyond the normative assumptions of ASIC enactment.

This article presents findings from three case studies (three universities) within an empirical research project exploring the process of ASIC and the subjective experiences of those driving the process of program implementation. It will reveal knowledge of the narratives of key actors, which can enhance the implementation of ASIC. This article outlines an overview of the relevant literature on the role that narratives in policy implementation play, and provides insights from FI theory. It critiques the role of ASIC in addressing gender inequality in the higher education sector, followed by a narrative analysis (Bischoping and Gazbo, 2016) and a discussion of key findings from the empirical study.

### Policy context for the Athena SWAN Ireland Charter

The Athena SWAN Ireland Charter (ASIC) framework is an extension of the UK Athena SWAN Charter. The awarding body of ASIC awards in Ireland is the statutory Higher Education Authority (HEA). In 2015, the HEA agreed to coordinate and fund the extension of the Charter to Irish HEIs. Advance HE, a UK-based member-led, sector-owned charity that works with institutions and higher education across the world to improve higher education for staff, students, and society, supports the roll-out of the Charter in Ireland. The ASIC has evolved since 2015 to support Irish HEIs and academic units to work toward impactful and sustainable gender equality actions and to build capacity for evidence-based equality work across the equality areas as enshrined in Irish legislation (Advance HE, 2022). Commitment to the Charter is a key pillar of Ireland's national policy drive for gender equality aligned with European Commission conditions for Horizon Europe funding, with progress connected to institutional eligibility for funding from Ireland's major research agencies.

A national consultation of Athena SWAN Ireland in 2021 identified the Charter-mark accreditation as a key driver of gender equality in Irish HEIs (Rothwell and Irvine, 2022; p. 5). Results from a survey of HEI staff indicated that ASIC provides staff with the necessary language to identify gender-based inequities and in raising awareness of such inequities. On the topic of the ASIC workload, respondents called for ASIC working time to be factored into workload allocation models, for more resources across the sector, and for easily accessible and nationally benchmarkable staff data. Mirroring concerns around the workload, the Athena SWAN Charter in the UK has been criticized for an uneven distribution of the workload within Charter self-assessment teams (SATs) falling to women (Caffrey et al., 2016). However, as Drew (2021, p. 33) notes in her review of the ASIC, this phenomenon is neither new nor specific to ASIC work, although it screams of irony, and solutions should be formulated “to incorporate such imbalances into Athena SWAN gender actions to address uneven allocations and their gendered outcomes.”

The normative assumption of the ASIC underpins the preparation of the application for an institutional or unit-level Athena SWAN award, whereby a self-assessment team (SAT) must be established. Athena SWAN Ireland states that the award and the implementation of the action plan within 4 years “depends on assuming a collective responsibility for addressing systemic inequalities and embedding inclusive cultures in higher education” (HEA, 2021). The HEA goes on to advise that the SAT is responsible for collecting and analyzing data, consulting with the community, developing and evaluating actions, and communicating findings, activity, and progress (*ibid*). The SAT should be representative of the staff profile within the institution. It is “not a review group. They should have authority to make decisions that will drive equality work. Their reporting line should reflect this status.” Advance HE (2017) is also explicit in their statement on the SAT that “it is not feasible that any one or two individuals be responsible for completing the whole application.” Essentially, the policy guidance is very clear—the SAT is a core agent in driving the change agenda formalized within each Athena SWAN Ireland action plan, which is implemented once the award is attained from the conferring body. Irish public policy discourse on ASIC implementation does not refer to the role played by varying perceptions and narratives of key institutional actors nor to the part they may play in ASIC sustainability and embeddedness. It is in understanding these narratives that a more contextualized process could be informed (Ní Laoire et al., 2020) since the Charter implementation is a highly context-sensitive process (Hodgins et al., 2022), despite its normative assumptions. The following section describes the theoretical framework created for the empirical study.

## Theoretical framework

A hybrid theoretical framework has been constructed for this study's inquiry in order to explore how to elicit meaning from policy actors' narratives in collectively implementing a gender equality program within men-dominated higher education institutions in Ireland. The theoretical framework, therefore, draws insights from the literature on the role of analyzing narrative accounts within the space of policy program implementation and insights from feminist institutionalism (FI).

Henderson and Bhopal (2021) study of the narratives of academic staff involved in the Athena SWAN and Race Equality Charters in the UK is illustrative of the role institutional actors play as policy translators, in embedding Charter marks in the higher education sector. The ways in which the key institutional actors tasked with embedding the Charter understand, perceive, and reconstruct the process as a whole are important, resonating with Ciccia and Lombardo's (2019) study on the transversal role of discourse and narratives in policy formulation. Legesen and Suboticki's study (2021) of academic department heads negotiating the contested pathway of enacting gender balance policies and Mannell (2014) study of narratives in framing gender as a policy issue, illustrate the ways in which narrative accounts highlight the role played by key actors' perceptions and interpretations in the implementation of policy recommendations, by undermining or facilitating the process. Therefore, by examining the narrative accounts, whereby “meaning is fluid and contextual, not fixed and universal” (Reissman, 1993; p. 15) of key institutional actors, we can better understand this contested policy space of gender equality program implementation. This article builds on feminist studies that highlight the role of discursive strategies in the framing of policy problems and individual and collective opposition as drivers in determining policy implementation (Mergaert and Lombardo, 2014; Cohen et al., 2020).

Joan Acker's study on inequality regimes (Acker, 2006) along with political scientists working in new institutionalist studies (in particular, Hall, 1986; March and Olsen, 1989; North, 1990) enabled feminist sociologists, feminist political scientists, and new institutionalist scholars to create feminist institutionalism (FI). The FI framework drawn on for the purpose of this article is built upon sociological institutionalism (Mackay and Waylen, 2009; Powell et al., 2018), an analytical framework for exploring the gendered nature of social and political institutions and their gendering effect on power dynamics and gendered outcomes (Mackay et al., 2010; Gains and Lowndes, 2014, 2018). Within FI theory, institutions are defined as comprising formal and informal rules, norms, and outcomes, and explanations of change are actor-centered (Schmidt, 2010; Bogaards, 2022). Lovenduski (2014) notes that FI highlights the gendered dimensions of structures of power and behavior, more than previous iterations of the new institutionalist theory were able to uncover. FI is concerned with analyzing the relationships between actors and institutions, unpacking their gendered interaction between formal and informal rules, practices, norms, and narratives, and exploring the impact of these dynamics on institutional power relations and broader gendered discourses (Krook and Mackay, 2011). FI theory asserts that efforts to introduce changes may be undermined by varying perceptions of the problem by key institutional actors (Hodgins et al., 2022). White and O'Connor (2017) argue that resistance to gender equality programs is normalized through discourses based on concepts of excellence, gender neutrality, and choice. Resistance can be mirrored in the framing of issues, thus, limiting the visibility of the problem and solutions (O'Connor, 2020). Gains and Lowndes (2018) argue that a focus on the role of institutional actors has the potential to uncover the dynamics and actor-centered narratives of gender equality reform, as well as explain policy outcome variation due to actors' interpretation in operationalizing an institutional framework. FI scholars with an interest in the role of the discursive nature of institutions explore how institutional structures and culture may influence the perceptions, interpretations, and narrations of key policy actors (Kulawik, 2009; Freidenvall and Krook, 2011). Often of interest to FI and feminist scholars, more generally, is the phenomenon that when working to tackle gender inequalities institutionally, some actors find themselves as ambivalent, particularly those working closest to the operationalization of implementation (Wieners and Weber, 2020), resonating with Meyerson and Scully's (1995) “tempered radicals.” This idea of actor's ambiguity in working to challenge the institutional status quo while implementing equality programs has also been elucidated from Ahmed (2012) study of diversity practitioners in UK and Australian universities. Her seminal study found that key institutional actors who are working to implement institutional equality change face the promise and pitfalls of “institutional happy talk,” whereby individuals in management/leadership roles consistently portray the institution in a positive light, with little variance in this messaging. Ahmed reflects that the “institutional happy talk” narrative is expressed and created by those in management/leadership positions to those “outside” the micro-level processes of implementation.

In HEI research conducted in Ireland, FI has been used to study the organizational structures and cultures of HEIs in order to uncover the dynamics between actors interfacing with institutional structures and cultural norms. O'Connor (2020) conducted an FI study of Irish HEIs, highlighting the importance of tackling organizational structures and culture in paving the path toward gender equality. Change has been slow in the men-dominated HEIs that resist change formalized within interventions designed to tackle gender inequality. Shining the FI lens on one Irish University with a history of legal challenges around gender inequality, Hodgins et al. (2022) examined the interventions/changes introduced to tackle gender inequalities, and how these interventions/changes were perceived by HEI staff. This study highlighted many factors consisting of institutional resistance to change, such as how many key institutional actors have limited understanding of cultural barriers to gender equality and gendered power structures; “they may therefore fail to understand why such intervention is necessary and may, in fact, actively resist change” (*ibid*, p. 18). The study findings warn against the danger of ASIC awards being the sole driver of structural and cultural changes because a multi-focal solution is required. The study highlights how staff predominantly perceived the university's interventions as failing to transform the organizational culture and structures meaningfully. This study importantly shines a light on the dissonance between public institutional discourse and the lived experiences of HEI staff, a gap between the “saying and doing” and rhetoric and practice (as also found in Cavaghan, 2017; Powell et al., 2018; Thomson, 2018).

A key weakness identified in Bogaards's (2022) review of FI is that the divergence of conceptual and empirical approaches has “stunted the development of feminist institutionalism (singular)” (*ibid*, p. 425), which has resulted in a lack of clarity as to how the various FI approaches and concepts are related. The review highlights the key strength of FI being that it brings together an analytical lens to meet the inquiring needs of feminist scholars and political scientists alike. This desire is to theoretically and empirically explore the gendered micro-foundations of institutions on adaptations to new processes with change interventions, such as the embedding of Athena SWAN Charter actions.

Insights on actor-related change, drawing from their exchanges with informal and formal institutional structures, from FI were chosen for this study because of its focus on the shaping effect of institutional rules and norms on individual actor behaviors, perspectives, and perceptions. This FI literature merged with my argument that narrative accounts provide crucial contextual knowledge about how ASIC eventually comes to be narrated and re-narrated by key institutional actors, as demonstrated in Henderson and Bhopal (2021) study, which creates the hybrid theoretical framework for the empirical study discussed in this article.

The following section outlines the research methods and analytical approach adopted for the empirical study.

## Materials and methods

A qualitative case study research design has been adopted for an empirical study involving three Irish universities, three cases, which have attained a minimum of the institutional Bronze Athena SWAN award. In order to explore cases with more tendencies for masculinist environments, universities alone were chosen for this study. The rationale being these institutions consist of the oldest, most prestigious, and most autonomous settings for higher education in Ireland (O'Connor, 2014), whereby this perceived status is linked to a tendency for men to occupy positions of power and prestige (Leathwood and Read, 2009). Cases have been purposively selected on the basis of their unique institutional equality context with selection criteria, including:

- The number of ASIC submissions made by the university;
- Universities that have had European Commission (EC) structural projects on the topic of gender equality;
- The longevity (age) of the universities;
- Public funded, National University of Ireland (NUI) (a federal university comprising constituent universities) and non-NUI institutions (independent universities);
- Universities that have Vice-Presidents (VPs) with an Equality role or VPs without this role/function;

This study received ethical approval from the Social Research and Ethics Committee (SREC), University College Cork, Ireland (Approval Number: 2019-048). Ethical approval for this study strictly prohibits the revealing of HEIs included in the study and personally identifiable information on research participants, including their position in the university, except when described in a general way. The rationale for this restriction is that the pool of staff working in Irish universities is relatively small and individuals could be easily identified by their position. Resonating with Ahmed's (2012, p. 10) logic for anonymity when she was interviewing university staff, anonymity created freedom to openly respond to prompts within the interview space without fear of potential workplace repercussions. Therefore, all efforts have been made by the author to balance the safeguarding of research participants' anonymity with the sharing of data findings in a detailed way as possible. Table 1 outlines descriptive information on the three cases.

**Table 1:** Research interview data on the three case studies.

	**Gender ratio of**	
	**research participants**	
	**Men (m)**	**Women (w)**
**University A. 10 interviews**	6	4
**University B. 9 interviews**	4	5
**University C. 7 interviews**	2	5

A total of 26 interviews were carried out with key institutional actors across the three cases (Table 1) between 2019 and 2021. These individuals are publicly committed to the work of implementing the ASIC process at an institutional university level. Public information on HEI SATS are available on university websites, which is where I started with the process of research participant selection. All were members of the institutional ASIC self-assessment team (SAT) at the time of interview. The research participants chosen for this study were purposively selected based on their employment role in the university in order to elicit a wide range of lived experiences, including participants from across academic, senior university management, professional, and research employment roles. The COVID-19 pandemic occurring in the middle of data collection meant that two (out of nine) interviews for University B and all seven interviews for University C were held online through Microsoft Teams.

### Narrative analysis of the data

Although defining the scope and nature of a narrative is a challenging and contested subject, Liu and Yu (2020) propose that the defining essence of a narrative is that it explains the link between events. This, in turn, is the main difference between stories and narratives—“if a story is the events of our day, the narrative is how we superglue them together” (Edmond and Bednarz, 2021; p. 27). In the empirical research narrative accounts explored and described in this article, the main events “supergluing” the narratives of the interviewed actors was the work they were carrying out and their perceptions around their involvement in the implementation of the ASIC locally. Research participants were asked to describe the ongoing process of their work in assessing and actioning the institutional ASIC. A semi-structured interview guide for the interviews with key ASIC institutional actors centered on three main areas of inquiry, framed by FI:

1. The perceptions, understandings, and experiences of key institutional actors engaging with the formal rules—actions as outlined in the institutional ASIC action plan—of program implementation.
2. The perceptions about working with the formal framework of ASIC within the context of informal institutional structures.
3. The nature and role of power relations in embedding ASIC actions.

Interview data were audio-recorded and subsequently transcribed. An interpretive narrative analytical methodology was used to elicit the narratives' accounts (Bischoping and Gazbo, 2016). This methodology is suitable for examining an implementation process that is complex (Ball, 2013). Several themes arose from the narrative analysis conducted on the interview data. The narratives were categorized thematically to develop six main narrative themes (Table 2), created through an iterative process between the literature and transcribed data. A similar approach was employed in Henderson and Bhopal (2021) study of the narratives of staff involved in the Charter marks in the UK. It was clear from the interviews that research participants communicated and wove a narrative based on their own subjective positioning and interpretations in implementing the Charter actions and in connecting the events of the implementation of the ASIC locally. Analyzing the narrative accounts and extracting the main narrative themes became the primary focus of analysis. This analysis enabled a nuanced understanding of the process of localized gender equality policy implementation.

**Table 2:** The main themes extracted from narrative analysis across the three case universities.

**Narrative themes**	**University A**	**University B**	**University C**
“I really don't think I'm the best person to interview”: Erratic employment of Charter staff and lacking operational knowledge of Charter implementation	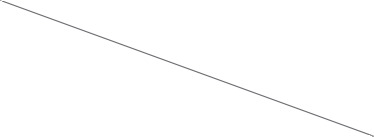	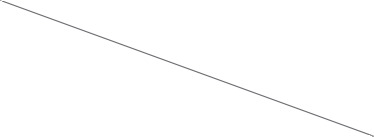	C 4 (m); C 1 (m)
“We need an Irish programme”—desire for a nationally contextualized Charter	A 1 (m)	B 6 (m); B 7 (w)	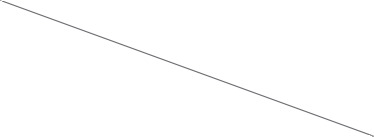
“It's good, but…”—Ambiguous narratives of the Athena SWAN process	A 2 (m); A 1 (m); A 3 (w)	B 3 (m); B 9 (m)	C 5 (w); C 6 (w); C 3 (w)
“It has a presence on all the big issues”—Supportive narratives of the Athena SWAN process	A 5 (w); A 9 (w)	B 1 (m); B 2 (w); B 4 (w); B 8 (w); B 5 (w)	C 2 (w); C 7 (w)
Narratives of “happy talk” about the Athena SWAN process	A 6 (m); A 8 (m)	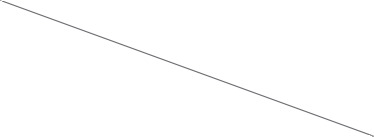	C 1 (m)
Narratives of identifying points of resistance that inhibits the Athena SWAN process	A 10 (m); A 11 (m); A 7 (w)	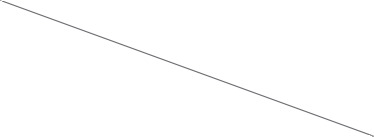	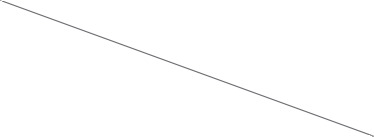

While engaging in the analysis, I was keenly aware of the positionings and roles of the research participants subjective narratives, resonating with the approach adopted by Wieners and Weber (2020). Most of the research participants have spent most of their working lives in the academy, as I have myself as an interviewer and data analyst. In conducting the interview and in examining the interview data, I was mindful of my positioning in the production and understanding of the narratives (Silver, 2013). I have been explicitly conscious through data collection and analysis of the role that I played as a woman researcher working in academia, and the role this could play throughout the research process, as it could account for the reactions of the research participants as reported in this article.

## Results

This section describes the six main themes extracted from the narrative analysis of interviews conducted with key actors working to implement the ASIC process at an institutional level. Narrative themes emerging as dominant from the data include a lack of operational knowledge, desire for a nationally contextualized program, ambiguity, championing, “happy talk,” and identifying points of resistance.

### “I really don't think I'm the best person to interview”: A lack of operational knowledge of Charter implementation

When contacting potential University C respondents for interviews, a striking feature of responses from people was that they were not comfortable being interviewed because they were not familiar with the detail of their institution's ASIC implementation. As one individual responded to me by email, a recurring response was along the lines of “I really don't think I'm the best person to interview.” Many individuals suggested that I speak with the person who had been working closest on ASIC work; however, this individual had resigned from the position and was not available for an interview. This explains why there are fewer respondents for this case than the others. A worrying pattern of erratic employment was also found from University C regarding the core work of ASIC implementation. The university had a pattern of hiring people for the intense workload of Charter applications and then letting staff go, as illustrated in this quote:

“And so we had this coordinator and she was really good. [] She was quite persistent. [] But she got it done and we submitted and we got it. So that was the application and then we let her go (laughs)” (C 1).

At the time of interviews, there was no leading person in post for ASIC implementation as was the case in Universities A and B. In fact, with Universities A and B, the main people working closest to the ASIC implementation knew the most information and detail when talking about the process. This reality jars with the idea that the entire ASIC SAT is a collective agent for change in the university (HEA, 2021). This study highlights the fact that operational knowledge of ASIC implementation remains with a few key people—or one person—and therefore staff turnover, as in the case of University C, can significantly disrupt the work being done and the institutional knowledge maintained.

### “We need an Irish programme”—desire for a nationally contextualized Charter

A theme that arose from the analysis was a desire by key actors to nationally contextualize the Charter to a greater extent than it had been or was perceived to be contextualized. At the time of interviews, the Charter had not been re-structured to be aligned with the national legislative and organizational context, as is the case today in Ireland (Advance HE, 2022). This theme affirms the necessity to contextualize gender equality programs to national contexts Ní Laoire et al.'s (2020). This study shows that key policy actors who are tasked to drive and implement such programs may not support a non-localizing implementation approach or be perceived as non-localized, as the quotations below illustrate from Universities A and B.

“I really would like to see nationally to develop our own Charter and it's not a nationalist thing either but just that in Ireland that we would develop a Charter.” (A 1).

A respondent from University B raised concerns about the idea that the Athena SWAN Charter in Ireland was being directed from the UK, and then later also stated that a more Irish-contextualized framework would work better because of differences between the Irish and UK contexts around behavior change in society generally.

“I worry about it because it's (Advance HE) a UK based organization and with Brexit to have Athena SWAN awards being factored by an organization head-quartered outside of the EU having a level of governance over EU funding is a concern. I think there are some learnings there of what generates behavioral change in our context which is a bit differently sometimes to a UK context so that would be my comment there” (B 6).

What was interesting with this theme was the perception by this senior member of staff that the Athena SWAN Charter in Ireland was ultimately being coordinated from the UK, which in reality is not the case. Another respondent in University B (B 7) stated that she felt the data-reporting systems were more amenable to the Charter application requirements in the UK, than was the case in Irish HEIs and has been highlighted in the literature on ASIC in Ireland (Drew, 2021).

### “It's good, but…”—Ambiguous narratives of the Athena SWAN Ireland Charter process

Ambiguity refers to a subjective experience of being open to more than one interpretation of an event, often felt as contradictory feelings toward an experience. This theme was dominated by ambiguity throughout, much in the way described by other researchers describing this phenomenon (Lagesen and Suboticki, 2021). When reflecting on the main events illustrative of their involvement in the Athena SWAN Ireland process (preparing for the application submission or preparing for implementing the action plan after receiving the award), participants engaged with this ambiguous theme qualified their statements throughout, with constant use of words such as “but,” “however,” and “yes, but” when describing their experiences. In this theme, participants were working in senior management, academic, and professional roles, with a gender balance in respondents. Dominant in this narrative theme are reflections on the good positive aspects of the ASIC process followed by a qualification of as to why it is not working as planned or hoped. When talking about a new career progression initiative initiated by the ASIC work, included in the institutional ASIC action:

“It does create some positive change but I've a love/hate relationship with it (ASIC) for various reasons (laughter). Some of it is to do with it not engaging sufficiently with various forms of equality and the other is just it is bureaucratic, demands... it's incredibly demanding in terms of the workload” (A 1).

This person spoke about how the Charter encouraged people across the institution to be aware of the gender equality topic and that ASIC has “helped things change culturally but I would say that those cultural norms have not been embedded in the regulations and policies of the University.” They also talked about the performativity of the Charter work itself and the enormous workload it entails, highlighting the gendered aspect, as has been found in previous research:

“Write the application in a way that's very specific to the requirements of the application template and it means that there's a high level of performativity in it, you end up having to negotiate it being an equality exercise and very much a technical writing exercise [] High workload may reproduce inequalities for others in that too.”

A person from University B spoke about the contradictions in addressing gender inequality when the problem of inequality is rooted in systemic legacies; and the respondent then goes on to talk in a more positive light about the ASIC:

“So if you formalize it all too much then you do lose the ability to gain genuine insights into people's ability. But it's the informal old boy networks that are at the root of an awful lot of the inequality that has been there traditionally so how do you square those two? But Athena SWAN provided a framework by which we could actually structure our engagement with gender equality.”

A respondent from University C refers to the ASIC as being more of a marketing or branding initiative as opposed to embedding change, and then goes on to talk about the positive impact the Charter is having on the HEI:

“At the moment it is a particularly identified brand which comes with it, lots of initiatives and activities, but that risks perhaps … this is only my own reflection … of it becoming an Athena SWAN thing [] as opposed to a deeply embedded university practice which doesn't have any other name other than the way we do things around here.

But I think Athena SWAN has had wide ranging impact into the organization [] surfacing issues which may have previously gone undiscussed or unhighlighted. [] I think there has been extensive opportunities for people to discuss equality and its more general sense (C 5).

These extracts from the interview text are illustrative of the tone and content of the narratives, which essentially are positive aspects to the Charter-mark policy work, and twinning challenges inherent also, reflecting a dissonance in the narrative account.

### “It has a presence on all the big issues”—Supportive narratives of the ASIC process

The main feature of this narrative was the positive messaging embedded throughout, resonating with previous research findings, whereby positive perceptions of the Charter were elicited, particularly from institutional champions, as are the respondents reporting in this theme (Hodgins et al., 2022; Rothwell and Irvine, 2022). Academic, senior management, professional, and research staff responded in this thematic category, the majority of whom were women bar one man. Overall, the narrative conveyed a message of positivity for the formalized process of ASIC, often with respondents referring to their own experience as the following illustrates:

“Athena SWAN now very much (is) on the radar and it does influence conversations and it does influence decisions that are being taken in a way that the issue maybe not could be ignored in the past but could be somewhat side-lined or quietened down a bit, whereas now that we are an Athena SWAN accredited University I think that by definition it has a presence on all the big issues and in terms of the direction and the format of the structure and the aims and the objectives of the University.” (A 5).

This narrative theme differed from the theme of “institutional happy talk” as described in the following section, in the sense that this supportive/championing theme involves the research participant detailing the work that they are doing, reflecting on their own work on the SAT, not in any way vague about their own experience. Within this theme, they had been working in the institution for most of their working lives, championing social justice issues institutionally as well as through their own research, as was the case with respondent A 5, who was working in this manner for 20 years in the same HEI.

This narrative theme examines the nature of the formalized process of the ASIC, and how its formalized process was, as respondents stated,

“Forcing the University to face up to longstanding historical gender discriminations, practices that failed to take into account the difference you know and we're getting closer toward... but we're nowhere near equality of outcome... but we've certainly shifted from equality of opportunity, you know that we're recognizing the difference and we're making slow progress but slow and steady I think” (A 5).

“So the school SATs would be kind of formalized, they're formal. We're not just relying on the informal… [] many people may be completely, you know, may not be fully engaged with it … whereas when it comes down to the local level, to the school level, not only then do they wrap their heads around the institutional application but then they have to look at ways they can build on it and they can do things locally …that's where I see the real benefit of Athena SWAN, is the schools (B 8).

These respondents explained their preference for the formalized framework of the Charter because of the accountability that comes with formality. This point about formality resonates with the tenets and assumptions of FI, revealing a narrative at the interface of an actor working with a formal process.

### “Narratives of “happy talk” about the Athena SWAN Ireland process”

The main features of this theme included the following: first, the respondents spoke vaguely or avoided a question about their own individual action in implementing Athena SWAN in the institution, referring to the work of others, and the work “those others” were doing—not themselves. Second, the respondents implicitly or explicitly spoke in a hyper-positive way about the Athena SWAN work, or in the words of one respondent when voluntarily reflecting on the corporatist nature of contemporary universities, the need for some senior members of staff to present “always this sort of manically positive face to the world (A 1).” Respondents included staff from senior management and senior professional roles were all men, highlighting the gendered dimension to this narrative theme in men-dominated Irish HEIs, who are working in key university decision-making roles. Contrasted with this were women senior managers who spoke in more detailed ways about the work they were doing on embedding ASIC actions (for example, University B). Ahmed (2012) refers to a brick wall analogy when describing the barriers diversity practitioners come up against when implementing diversity programs in the HEI, oftentimes with senior management. This analogy of the “brick wall” was relatable for me as an interviewer in experiencing and then analyzing this narrative theme. Connected to this was the idea that the reputation of the university is important. This was how it was experienced by myself to conduct these interviews that resulted in the “happy talk” narratives; witnessing the creation and practice of a positive face of ASIC to the world. The following extract illustrates a respondent avoiding a question pertaining to their direct action on the SAT:

Interviewer: Yeah. Okay, so question …, keeping in mind the work you do as part of Athena SWAN can you tell me how it works with existing processes? So whatever you're directly working on, if you keep that in mind.
Respondent: Okay. Well I mean firstly I think one of the most important things for... to give I suppose truth to Athena SWAN and the principles we're trying to give effect to is you need evidence so certainly from (name of unit) perspective we've put in a huge commitment to improve our data sets and we've invested significantly in resources, both people and both in developing new ways of doing business.

What was interesting about the conduct of interviews with respondents reflecting a “happy talk” narrative about Athena SWAN work was their vagueness way of speaking (as shown in the above extract), and there were no specific examples given, thus defining the vagueness or non-answering.

A respondent in University C spoke very positively about the ASIC process in his university:

“What I like about Athena SWAN is that it's … you critically analyse yourself, right. So it's self-analysis and self-reflective. I still think one of the most important things in all of this is heightening awareness. So to make … because also, you know, a lot of discrimination is subliminal. It's not overt. Well some of it is overt, we know, but there is other bits of it which are subliminal and people not thinking” (C 1).

However, when asked to complete a visual mapping exercise that involved respondents specifying the Charter tasks they have responsibility for or leading on (as is required of SAT members), mapped out by themselves on a piece of paper, his demeanor became defensive, he then stated that most of the functions are being carried out by others (in professional roles); he did not complete the task and said he would send on by email, which did not happen.

Wieners and Weber (2020) found that narrators positioned within the organization working in management/leadership positions were much more comfortable and experienced at verbalizing a particular impenetrable “manically positive” narrative of their institution. This resonates with this theme. Overall, this theme illustrates both the vagueness of the work the participants are doing, plus exemplifies the assertion of positive messaging of the ASIC implementation to an “outsider.” The gendered nature of an impenetrable institutional happy talk is a fascinating dimension to this finding.^1^ It resonates with the gendered nature of ASIC work across the cases, whereby women participating in the research worked on the daily operationalization of ASIC work, starkly contrasted with mainly men in senior management roles engaging in “institutional happy talk.” This finding also echoes Hodgins and O'Connor's (2021, p. 1) study of gender equality in an Irish university, specifically “the intractability and covertness of men's power and privilege,” and deserves further inquiry in future research. The finding is important as it highlights the messaging being portrayed by those in management/leadership positions may be detached from the actual operationalization of the ASIC work, as well as raising awareness of the tendency of a genderedness of this “institutional happy talk.”

### Narratives of identifying points of resistance that inhibit the Charter process

Resonating with research on resistance to gender equality initiatives' implementation in Ireland (Hodgins et al., 2022), a theme was developed that identified points of resistance within narrative accounts of the ASIC process without balancing this messaging with talking about what was also positive about ASIC, as with the ambiguous theme.

Reflectors of this narrative worked in academic, research, and professional roles in University A, two men and one woman responding. Characteristic of responses in this theme was also a degree of frustration with identified resistance—frustration with the homogenous nature of the men-dominated institution and with decision-making procedures in the university that are mirrored in the ASIC process. No person was working in a management/leadership role portraying and communicating this wariness narrative, resonating with research findings from Wieners and Weber's (2020) and Striedinger's (2017) studies, although participants engaging in this theme were critical of senior management's role in maintaining the status quo through institutional resistance.

This narrative theme is characterized by participants who were discussing and identifying points of resistance that shaped their perception of the ASIC process. An example of this identification of resistance to implementing ASIC was in one participant's response about the lack of meaningful input to the decisions on actions made, and the decisions on the types of actions included in the institutional action plan.

“Like I know of plenty of staff within (this institution) that have maybe an issue close to their hearts, that got involved in Athena SWAN, that are pushing that forward, that are championing their area or position, you know myself would be included. But again at the end of the day the decision will be made by those who have to I suppose enact those actions and you know I can certainly see a scenario whereby someone with a lot of responsibility in (name of institution) looks at one action and goes “If I do this my life is going to get a lot harder and if I do this other one it's fine, it's easy, and it'll have some benefit” (A 11).

This participant referred also to “a lack of transparency on decision making processes for Athena SWAN work.” The intriguing aspect of this theme was that the subjective experience is portrayed as they (A 11), as a member of the core institutional committee, see themselves as separate from the people who will “enact the actions.” This conflicts with the discourse around the work of Athena SWAN nationally, whereby there is no mention of members who may feel less than equal or separate from others on the SAT participating in the decision and actions of the Charter implementation.

This theme highlighted a lack of certainty around the way of working with implementing the ASIC program locally and institutional resistance to Charter implementation. The below extract illustrates this point of the lack of certainty, specifically about commitment to the Charter mark being a non-performative (Ahmed, 2012) around the work being done and the institutional resistance being highlighted:

“You eventually get some commitment to fund it that increases the chance that it will happen but still it doesn't mean it's going to happen and we've had this happen. I mean we've had explicit examples of this happening where they make a financial commitment and then they don't... because nobody is responsible for delivering on the action nothing happens you know so. I spend a lot of time thinking about well what happened in that meeting and why did we make no progress?” (A 10).

Common throughout these narrative accounts was a critical perspective of the most senior management in the institution, often referred to as ‘they.' This extract illuminates a lack of understanding of meritocracy, illustrating institutional resistance (Hodgins et al., 2022) in the university when talking about the role of senior management in the work:

“There is a lot of resistance to this work you know. There is a lot of resistance to this work. There's a lot of resistance because you know the idea of meritocracy, oh, surely if you are merited you will get... I... sure... I think yes but let's not forget that there are all sorts of other issues.

You know and then there's all this discussion about “oh it's a … universities are a meritocracy,” I mean trying to get people to understand that it's not, you know that's also very complicated because people come with all sorts of biases, right” (A 10)

This narrative theme also raised questions about the ability to meaningfully deliver on the gender equality Charter given the male dominance and homogeneity of the institution's workforce, particularly in senior management/leadership roles.

“But if you actually do a survey of all (people in) all universities (working on equality issues). I'm sorry to say this but they all look like (name of person leading equality agenda in the university- a man). They all look like him (laughter). Why? And we laugh but why is that? Do you see what I mean? And I find that it's reflecting homogeneity” (A 10).

This theme highlights the perceptions of key Charter actors when they identify points of resistance that inhibit the Charter process. This resonates with awareness of institutional resistance to gender equality program implementation in previous similar research (Powell et al., 2018; Hodgins et al., 2022).

## Discussion

I have argued in the previous section that the themes derived from narrative analysis provide important contextual insight into the implementation of the Athena SWAN Ireland Charter. This is the first time that research conducted on the Irish Charter experience has been carried out on universities using the unique hybrid theoretical framework. In this way, the study's findings contribute to the literature on embedding structural gender equality programs in higher education, as well as to the ongoing process of Charter implementation in Ireland. In order to discuss these insights further, I will explore the six themes alongside the implementation of the ASIC at a national level, as described earlier in the article.

As seen in Table 2, there was a convergence of the themes of ambiguity and championing across the three cases. University C, with a history of erratic employment for ASIC-specific personnel, diverged as the only case to report a theme around a lack of Charter operational knowledge. University A diverged as the only case to report a theme of identifying resistance to ASIC implementation around the process of ASIC. In fact, University A was the only case reporting themes of happy talk and identifying resistance, and this dissonance reflects a gap between rhetoric and practice, as Cavaghan (2017) highlighted in her study of embedding gender mainstreaming programs. This phenomenon has also been found in previous Irish-focused FI studies (O'Connor, 2020; Hodgins et al., 2022). Rather than concluding that University A is unique with this dualistic thematic patterning, it is more likely that University C's lacking operational knowledge and University B's unique history institutional equality meant that more explicit dissonance did not arise from the latter cases. Also, interviews with University A participants were all in-person, whereas they were mostly online with Universities B and C, which may also impact the resulting findings. Both Universities A and B reported the desire for an Irish-specific program to be developed and adapted to the UK Athena SWAN Charter framework. Some of these individuals had previously worked in UK universities which may explain their positioning.

This article sought to gain a deeper understanding of the reality of the policy implementation process which is underpinned by the normative assumptions of ASIC enactment, namely that the SAT is the core agent for change working collectively and equally, with work not falling to one or two members of the team. This study has shown across the cases, highlighted by University C, that work, and knowledge, remain with a few key people, which makes the ASIC work vulnerable to staff turnover. Implied within the normative understanding of ASIC implementation is that the institution acts and responds within a unidimensional Charter mission and goal, whereas this study across all themes except for the supportive/championing theme highlights the gap and dissonance between public institutional discourse and the lived experiences of HEI staff, a gap between the “saying and doing” and rhetoric and practice. This has also been reiterated in previous Irish HEI research (Hodgins et al., 2022) and across the sector more generally (Cavaghan, 2017; Powell et al., 2018). Therefore, in order to ensure ASIC sustainability and embeddedness, as is the goal of the Charter in Ireland, more localized dialog and discussion are needed institutionally in paving the way for implementation complexity and nuance (Ní Laoire et al., 2020).

In answer to the question posed in the introduction—could our understanding of the narratives of key institutional actors play a role in enhancing ASIC implementation?—this article has shown that a greater understanding of the perceptions of institutional actors is integral to the embedding of gender equality within universities studied through the Athena SWAN Ireland Charter, especially as this research is the first of its kind in Ireland. Literature on the role of narrative accounts highlighting a diversity of perceptions in policy and program implementation is strengthened by this study's findings. All six themes highlight and uncover varying perceptions of central institutional actors in ASIC implementation. In particular, the narrative theme calling for Athena SWAN to be nationally contextualized resonates with Ní Laoire et al.'s (2020) argument of the importance of the constitutive role played by local and national context in the interpretation and implementation of gender equality program policy in higher education, as well as highlighted the variance in understandings and perceptions of the ASIC, as Hodgins et al. (2022) also found. Overall, this study resonates with similar previous research, in Ireland and beyond (Henderson and Bhopal, 2021), that highlights the importance of varying subjective perceptions and understandings in the implementation of an equality program, thus providing insight into the further sustainability and embeddedness of the Athena SWAN Ireland Charter.

FI informed this study's inquiry in order to allow an exploration of key actors' perceptions as they reflected on their work in implementing ASIC in their local HEI context. In the interview spaces, research participants were asked about their perceptions and experiences in engaging with the formalized ASIC process, perceptions about working with the formal framework of ASIC within the context of informal institutional structures, and the nature and role of power relations in embedding ASIC actions. The findings uncovered a diversity of perceptions with some gendered patterns, most notably the dominance of men reporting in the theme “happy talk.” In men-dominated HEIs (O'Connor, 2020), this finding is unsurprising and resonates with similar research in other countries and contexts (Ahmed, 2012; Mergaert and Lombardo, 2014; Powell et al., 2018). The theme of identifying resistance in the implementation of ASIC was notable in elucidating reflections on perceptions and experiences of resistance from central actors enacting Charter actions, resonating with FI literature that highlights the role of institutional resistance as undermining the roll-out of the gender equality program (*ibid*). The ASIC workload was raised in data findings (ambiguity theme). There was a clear gendered pattern across individuals who carried out the operationalization of ASIC implementation work as being women (Wieners and Weber, 2020). Using FI as the frame for interview schedules and narrative analysis as the conceptual and methodological lens, this study contributes to the literature on FI with a specific focus on the role of discourses and narratives (Freidenvall and Krook, 2011; Thomson, 2018).

Although not a comparative study across international contexts, this study resonates with Tzanakou et al. (2021) study of two of the main gender equality schemes used by research-performing organizations in Europe: Athena SWAN (UK) and Total E-Quality Award (Germany). The study found strengths in terms of the ability of these accreditation systems to drive gender equality and diversity within higher education institutions with suitable self-assessment tools that “encompass (an) intention to improve and advance through progressive approaches and renewals/re-audits, rather than simply assessing achievements in the past” (*ibid*, p. 6). This identified strength resonated with the research participant's perspective on Athena SWAN Ireland Charter as a means to drive change within the institution (supportive/championing theme). A common theme across the implementation of the Charter program across country contexts is the gendered nature of Charter work. Tzanakou et al. (2021) found that the burden of Charter implementation—the workload associated with it—continues to fall on women, mirroring other studies' findings (Caffrey et al., 2016; Ovseiko et al., 2017; Tzanakou and Pearce, 2019) and confirmed in this research study also. Nash et al. (2021) conducted a study exploring the perceptions of gender equity among SAGE Athena SWAN self-assessment team members in an Australian university, reflecting on the potential translation of Charter work into unequal gendered workload distribution, and thus undermining the potential impact of the Charter in addressing gender inequality in the university. This reality of Charter work causing an unequally gendered impact needs to be highlighted at the start and throughout Charter work planning as it raises awareness of the issue and push back against its occurrence.

There were several limitations in this research study. Unfortunately, students were not included in the interview sample, as none were available at the time of the interviews. Not having the student experience in the study means that the perspectives derived from the overall participant sample is lacking a holistic representation of the institutional SATs. The COVID-19 pandemic occurring in the middle of data collection limited the study in the sense that some interviews for University B and all for University C were held online, which was a limitation in the study given the non-standardized data collection methodology across the dataset which may have given rise to differing results, as well as a sense on my part as an interviewer of a decrease in rapport formed with respondents. Also, given that there are currently 19 institutions with ASIC awards in Ireland (Advance HE, 2022), the findings of this study cannot be taken to generalize the experience in Ireland but rather shine a light on understandings of narratives informed within subjective perceptions and positionings of institutional actors central to ASIC implementation.

The Minister for Further and Higher Education in Ireland states that “Athena SWAN is one of the most important initiatives that we have in the higher education sector in Ireland (Advance HE, 2022).” The Charter framework is an integral pillar to the implementation of gender equality in Irish HEIs, through structural and cultural transformations (HEA, 2018; Advance HE, 2022). Overall, the implementation of ASIC is received positively by surveyed respondents (Rothwell and Irvine, 2022). However, limited research in this area in an Irish context has highlighted a dissonance between HEI staff perception of gender equality initiatives generally (O'Connor, 2020; Hodgins et al., 2022), thus highlighting the need for an empirical study as described and discussed in this article, which has been informed by literature on the role of narratives in policy program implementation and FI (O'Mullane, 2021).

This study, for the first time in Ireland, explores perceptions of key institutional actors who are tasked with implementing ASIC locally within their HEI. Overall, the findings from the empirical research study are significant to the cause of addressing gender inequality in HEIs in three main ways. First, they demonstrate the importance of the “cultural understanding of equality,” as coined by Hodgins et al. (2022). Findings showed that men in senior positions in HEIs were not able to specify ASIC actions they were engaged with themselves, apart from oversight and speaking of the equality agenda generally in the institution. Failure to understand the depth of detail ongoing within the ASIC process could lead to such central senior decision-makers failing to holistically understand the scope of actions, as was also shown in Hodgins et al.'s (2022) study. Second, connected to this latter point, findings highlighted the dissonance between “saying” about ASIC at a national public level—for instance, the SAT is a collective agent whereby all members contribute meaningfully to the implementation of actions—and the reality that, based on this study, there exists a core group within HEIs knowledgeable about the implementation process. As was shown in University C, this leaves the work vulnerable to staff changes which can in turn endanger the sustainability of the work. Third, the study reveals the gendered nature of ASIC implementation, as seen notably in the more detached and vague narratives of senior men with women operationalizing the Charter work locally. Excavating deeper into the feminist institutionalist nature of ASIC implementation with a sole FI lens in further research on this dataset and future research would uncover the dynamics of gendered power relations. Research findings provide vital insight into the varying perceptions and experiences of institutional actors central to implementing Athena SWAN in Ireland, providing theoretical learnings and practical knowledge for further sustaining and embedding the Charter framework in HEIs going forward. Embedding gender equality in higher education is a context-sensitive process, which benefits from progressing at an incremental pace rather than rushing to meet a requirement and tick a box.

## Data availability statement

The datasets presented in this article are not readily available because they contain confidential identifiable data. Requests to access the datasets should be directed to MO'M, m.omullane@ucc.ie.

## Ethics statement

The study received ethical approval from the Social Research and Ethics Committee (SREC), University College Cork, Ireland (Approval Number: 2019-048). Written informed consent to participate in this study was not required from the participants in accordance with the national legislation and the institutional requirements. Written informed consent was obtained from the individual(s) for the publication of any potentially identifiable images or data included in this article.

## Author contributions

The author confirms being the sole contributor of this work and has approved it for publication.

## Footnotes

^1^Many thanks to Reviewer 2 for their comments regarding this finding.
